# Artificial Intelligence Accurately Detects Traumatic Thoracolumbar Fractures on Sagittal Radiographs

**DOI:** 10.3390/medicina58080998

**Published:** 2022-07-26

**Authors:** Guillermo Sánchez Rosenberg, Andrea Cina, Giuseppe Rosario Schiró, Pietro Domenico Giorgi, Boyko Gueorguiev, Mauro Alini, Peter Varga, Fabio Galbusera, Enrico Gallazzi

**Affiliations:** 1 AO Research Institute Davos, 7270 Davos, Switzerland; boyko.gueorguiev@aofoundation.org (B.G.); mauro.alini@aofoundation.org (M.A.); peter.varga@aofoundation.org (P.V.); 2 Department of Orthopedic and Trauma Surgery, University Hospital Basel, 4031 Basel, Switzerland; 3 IRCCS Istituto Ortopedico Galeazzi, 20161 Milano, Italy; andrea.cina@grupposandonato.it; 4 ASST GOM Niguarda, 20161 Milano, Italy; giusepper.schiro@gmail.com (G.R.S.); pietro.giorgi@ospedaleniguarda.it (P.D.G.); 5 Spine Center, Schulthess Clinic, 8008 Zurich, Switzerland; fabio.galbusera@kws.ch; 6 UOC Patologia Vertebrale e Scoliosi, ASST Gaetano Pini-CTO, 20161 Milano, Italy; enrico.gallazzi@gmail.com

**Keywords:** vertebral fracture, fracture detection, heatmap, machine learning, artificial intelligence

## Abstract

*Background and Objectives*: Commonly being the first step in trauma routine imaging, up to 67% fractures are missed on plain radiographs of the thoracolumbar (TL) spine. The aim of this study was to develop a deep learning model that detects traumatic fractures on sagittal radiographs of the TL spine. Identifying vertebral fractures in simple radiographic projections would have a significant clinical and financial impact, especially for low- and middle-income countries where computed tomography (CT) and magnetic resonance imaging (MRI) are not readily available and could help select patients that need second level imaging, thus improving the cost-effectiveness. *Materials and Methods*: Imaging studies (radiographs, CT, and/or MRI) of 151 patients were used. An expert group of three spinal surgeons reviewed all available images to confirm presence and type of fractures. In total, 630 single vertebra images were extracted from the sagittal radiographs of the 151 patients—302 exhibiting a vertebral body fracture, and 328 exhibiting no fracture. Following augmentation, these single vertebra images were used to train, validate, and comparatively test two deep learning convolutional neural network models, namely ResNet18 and VGG16. A heatmap analysis was then conducted to better understand the predictions of each model. *Results*: ResNet18 demonstrated a better performance, achieving higher sensitivity (91%), specificity (89%), and accuracy (88%) compared to VGG16 (90%, 83%, 86%). In 81% of the cases, the “warm zone” in the heatmaps correlated with the findings, suggestive of fracture within the vertebral body seen in the imaging studies. Vertebras T12 to L2 were the most frequently involved, accounting for 48% of the fractures. A4, A3, and A1 were the most frequent fracture types according to the AO Spine Classification. *Conclusions*: ResNet18 could accurately identify the traumatic vertebral fractures on the TL sagittal radiographs. In most cases, the model based its prediction on the same areas that human expert classifiers used to determine the presence of a fracture.

## 1. Introduction

The thoracolumbar (TL) spine is the most frequent site of traumatic fracture occurrence [1,2], with blunt trauma being the most common cause [3,4]. Traumatic TL fractures are serious injuries associated with decreased physical function, severe reduction in the quality of life, and the lowest rate of return to work among all major organ injuries [2,3,4,5,6]. Although controversy exists, plain radiographs of the spine are commonly the first imaging step performed in trauma routine imaging, especially in hemodynamically stable patients and in low- and middle-income countries with limited availability of computed tomography (CT) and magnetic resonance imaging (MRI) [7,8,9,10,11,12]. Despite its current widespread use, the reported false-negative rates in diagnosing TL fractures on plain radiographs remain high, ranging from 24 to 67% [13,14,15,16]. A missed vertebral fracture can result in chronic pain, deformity, and delayed injury to the spinal cord and/or adjacent nerve root, which occurs in 19–50% of the cases [14,17].

The recent explosion of using labeled data, namely ‘big data’, has brought upon the era of artificial intelligence (AI) into the field of medical diagnostics and imaging, which has particularly benefitted from the application of AI based innovations [18]. Regarding imaging of the spine, promising results in the assessment of degenerative disorders [19], adult deformities [20] and adolescent idiopathic scoliosis [21], as well as in the detection of primary and secondary bone tumors [22,23], and vertebral fractures [24] have been recently published. Deep learning (DL) is a machine learning method that uses an algorithmic structure most commonly based on neural networks, such as convolutional neural networks. This method has been reported to perform equally well or even better than humans in image classification [25]. The power of this technique lies in the ability to identify and extract relevant features from labeled data at a grand scale [26].

The main aim of this study was to adapt existing DL models to accurately detect vertebral fractures on sagittal radiographs of the TL spine. The secondary aim was to gain a deeper understanding of the model’s interpretation of the “fracture zone“ through a heatmap representation. This study did not aim to compare the diagnostic accuracy of the DL models against expert human classifiers such as surgeons or radiologists. By supporting the treating physician in identifying fractures in the routine trauma imaging, the implementation of a diagnostic aid tool is anticipated to reduce the rate of missed vertebral fractures in plain radiographs.

## 2. Materials and Methods

### 2.1. Patient Selection and Image Acquisition

Imaging studies of 362 patients older than 12 years and treated for traumatic vertebral fractures from 2010 to 2020 in a Spine Surgery Reference Center (ASST Grande Ospedale Metropolitano Niguarda, Milano, Italy) were retrospectively reviewed. To identify the patients, internal disease and surgical codes corresponding to traumatic injuries of the spine were used (Table 1).

Fractures resulting from mechanisms other than trauma such as osteoporosis or pathologic fractures were excluded. After exclusion, only patients with complete imaging studies, defined as having a plain sagittal radiograph, CT and or MRI data, were included. By applying these criteria, 151 patients were selected for the final analysis.

**Figure 1:**
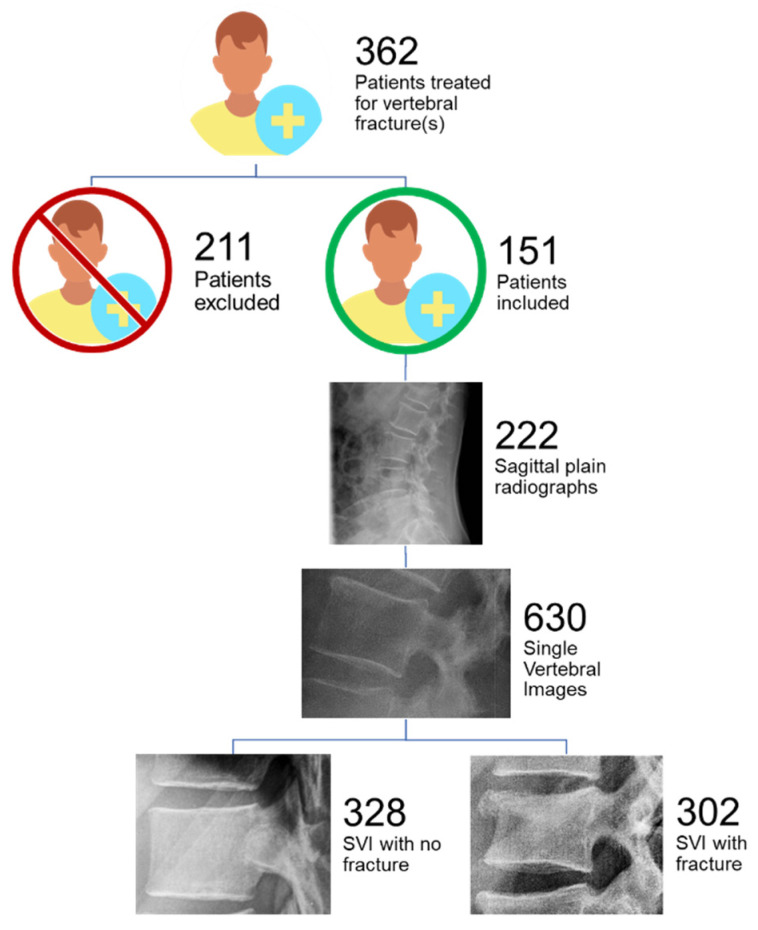
Patient inclusion and image set acquisition. SVI: single vertebral images.

In total, 222 sagittal radiographs of the TL spine and their corresponding CT and/or MRI data were obtained from the 151 patients. In case of repeated X-rays of the same patient with a change in the observed fracture morphology, more than one sagittal projections from the same patient was used, thus resulting in 222 radiographs from 151 patients (Figure 1).

### 2.2. Standard of Reference

An expert group of three spinal surgeons with more than 20 years of accumulated experience (approx. 15, 6, and 5 years) identified fractures on each sagittal radiograph, using all available image modalities to ensure a high diagnostic standard, including bone windows on Multidetector CT, low dose spine CT, and short tau inversion recovery (STIR) modality for MRI. The fractures where then classified according to the AO Spine Classification. Initially, each separate case was evaluated individually by each surgeon. For cases where disagreement existed, meetings were held to reach unanimous consensus.

### 2.3. Image Processing and Annotation

Each sagittal radiograph was annotated using C++ software code specifically developed for this study. According to the surgeon’s indications, the annotator found all fractured vertebrae present on each sagittal radiograph. Each fractured vertebra was cropped to produce a single vertebra image. This resulted in 302 single vertebral images classified as “fractures”. The vertebral level from T1 to L5 and the fracture type according to the AO Spine Classification were then assigned to the image. Further, radiologically confirmed non-fractured vertebrae corresponding to the same spine segments of the fractured vertebrae (e.g., thoracal or lumbar) were cropped from the same radiographs and included in a control group. To keep the groups balanced, 328 single vertebral images classified as “non-fracture” were cropped for a total set of 630 single vertebra images.

### 2.4. Adapting the Deep Learning Model

To achieve our aim, we first pre-selected, adapted, and then compared the performance of two existing DL models in the task of identifying traumatic fractures on plain sagittal radiographs of the TL spine.

**Figure 2:**
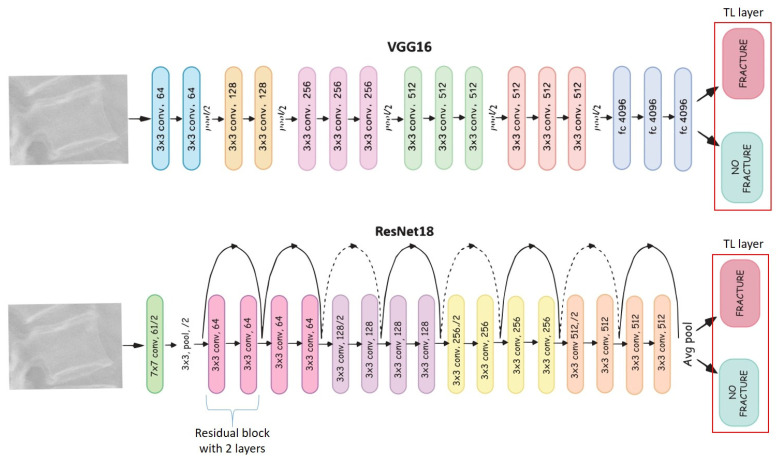
Comparison of the two deep learning convolutional neural model architectures VGG16 and ResNet18. Each colored block corresponds to a layer. The “fracture” and “no fracture” blocks are the output neurons. The last original layer of both architectures is removed and replaced by a layer with two neurons, namely “fracture” and “no fracture”. This technique of replacing the last layer of each network is called transfer learning. The dotted lines indicate an increase in the number of convolutional filters in residual block’s input to match the number of the output’s filters of the same block. TL: thoracolumbar; conv.: convolution.

Two deep learning convolutional neural network models VGG16 [27] and ResNet18 [28] were pre-selected as building blocks due to their state-of-the-art performance on computer vision tasks such as image classification, object detection, and landmark localization [29]. The main difference between the two models is that VGG16 is a plain neural network where the image is compressed step-by-step until the final classification layer, whereas ResNet tries to preserve, as much as possible, the input of each block using skipping connections (indicated by the arrows in Figure 2).

Since the number of available images was not high, we used transfer learning [30,31]—a technique where a model trained on one task will be repurposed to perform a second related task using the acquired knowledge of the first one. Namely, the two last residual blocks of ResNet18 and the final classification block of VGG16 were repurposed to the new task of vertebral fracture classification. A cross-validation with 10 folds was performed. The adaptations were implemented in Python language using PyTorch (Version 1.7, manufactured by Meta, Menlo Park, CA, USA) [32].

### 2.5. Training and Test Sets

The single vertebra image dataset was split into a training set (*N* = 578) and a test set (*N* = 52), both containing a balanced mix, namely, 278 “fracture” and 300 “non-fracture” single vertebra images in the training set, and 28 “non-fracture” and 24 “fracture” images in the test set. To increase the generalization capability of the model, we used augmentation techniques such as random rotation, flipping, and shifting. This allowed for training of the model on different versions of the same single vertebra images during the training epochs. The images were resized to 512 × 512 pixels and normalized to have zero mean and unit variance, according to the image guidelines used in the ImageNet challenge—a reference standard for computer vision tasks.

For training and evaluation we used a Linux workstation with a NVIDIA QUADRO RTX 5000 (Salt Lake City, UT, USA). The models ran for 200 epochs using a batch size of 32 and a learning rate of 0.00016. We used the Adam optimizer, a Pytorch classificator for model optimization and a method that reduced the learning rate by a factor of 0.1 if the accuracy did not improve for 10 epochs in a row (ReduceLROnPlateau in PyTorch). The model was implemented using the PyTorch library and the results and statistical evaluation were computed in the numpy and scikit-learn libraries.

### 2.6. Model’s Performance Parameters

The model’s performance was assessed quantitatively by calculating the accuracy, sensitivity, and specificity in fracture identification. Accuracy represents the ability of the model to assign the images to the correct class [33]—in this case, to predict the presence of fracture in each single vertebra image. Sensitivity describes the ability to detect fractures, and specificity is the ability to detect lack of a fracture.

### 2.7. Understanding the Model’s Prediction

To ensure that the model’s prediction was based on correct identification of the fracture zone, we conducted a heatmap analysis based on Activation Maps. These depict the areas of the image that led the model to classify the vertebra as “fracture” or “no fracture” by displaying a “warm zone”. Technically, they were obtained by multiplying the second last layer of the neural network by the weights that point to the neuron of the class predicted by the model. Finally, the same surgeons that set the standard of reference evaluated each heatmap to determine whether the “warm zones” correlated with the fracture zones seen in the CT and MRI data.

## 3. Results

### 3.1. Epidemiological Distribution of TL Fractures

**Figure 3:**
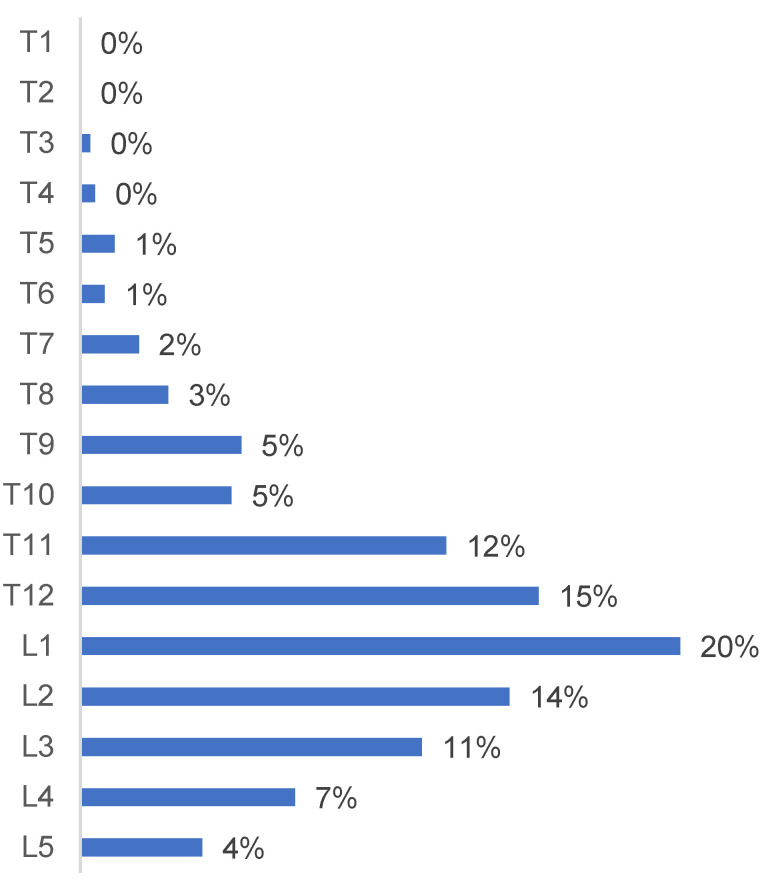
Epidemiological distribution of the thoracolumbar fractures at vertebral levels from T1 to L5.

**Figure 4:**
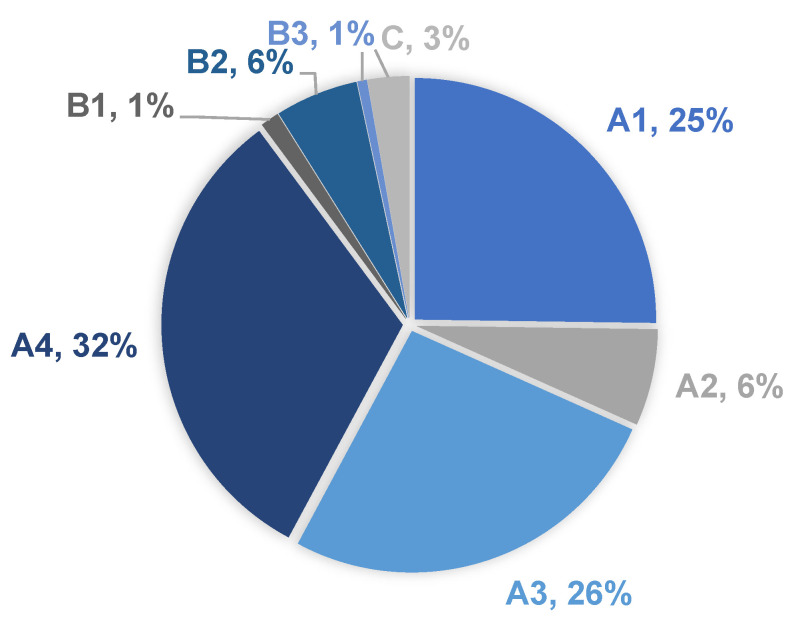
Thoracolumbar fracture types according to the AO Spine Classification and their distribution among the patients.

Vertebrae T12 to L2 were the most frequently involved, accounting for 48% of the fractures (Figure 3). A4, A3, and A1 were the most frequent fracture types according to the AO Spine Classification (Figure 4).

### 3.2. Deep Learning Model Performance

**Figure 5:**
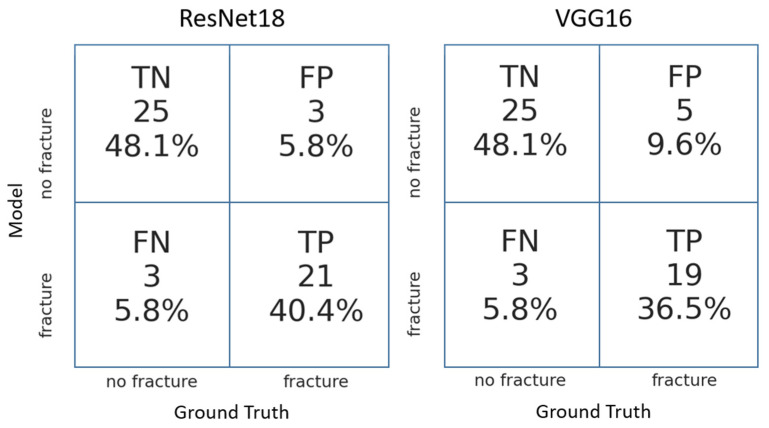
Confusion matrices obtained with the two deep learning convolutional neural models ResNet18 and VGG16. TN: True negative; FN: False negative; TP: True positive; FP; False positive.

**Figure 6:**
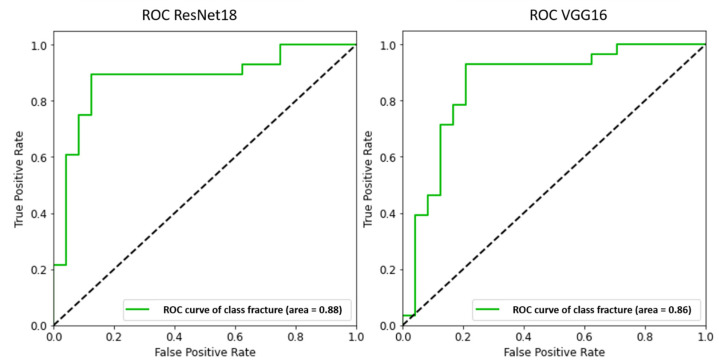
Comparison of the receiver operator characteristic (ROC) curve obtained with the two deep learning convolutional neural models ResNet18 and VGG16.

Both DL models achieved high accuracy, sensitivity, and specificity after hyperparameter optimization. In the direct comparison, ResNet18 displayed a better performance, achieving higher sensitivity, specificity (Figure 5, Table 2), and accuracy (Figure 6, Table 2).

### 3.3. Heatmap Analysis

**Figure 7:**
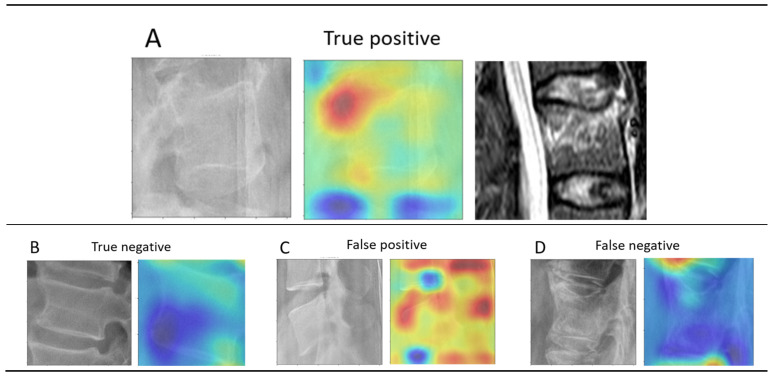
Heatmap analysis of the fracture zone. (**A**) Although challenging to observe on the radiograph (left), the signal hyperintensity in the MRI image (right) correlates with the “warm zone” on the activation map (middle). (**B**) No “warm zone” is displayed, thus ruling out the presence of a fracture. (**C**) Multiple “warm zones” are displayed, thus incorrectly suggesting presence of fracture(s). (**D**) No “warm zone” is displayed within the vertebral body, incorrectly ruling out the presence of a fracture.

In 81% of the single vertebrae images, the “warm zone” correlated with the fracture zone observed in the corresponding CT or MRI data (Figure 7). In the remaining 19% of cases, the “warm zone” was allocated to the immediate vicinity of the fracture zone.

## 4. Discussion

This study demonstrated that existing DL models can be adapted to accurately detect vertebral fractures on sagittal radiographs of the TL spine. Both models achieved similar sensitivity and specificity to that reported for expert surgeons and radiologists [24,34,35,36,37,38,39], however, our results should not be extrapolated to a human versus a machine situation given the lack of a strict comparison methodology. ResNet18 demonstrated better performance regarding the fracture identification task. A reason for this could be that the skipping connection aims to preserve as much information of the original image as possible, whereas VGG compresses the original image layer-by-layer in a sequential way, thus sacrificing the original input information. Additionally, ResNet18 was less resource intensive in terms of used memory (43 MB versus 524 MB) and faster in the inference. To our knowledge, this is the first study utilizing and adapted version of ResNet18 on the task of fracture identification.

### 4.1. Heatmap Analysis

In 81% of the cases, ResNet18 predictions were related to the regions on the vertebral body corresponding to fracture zones observed in the CT and MRI data. Although this finding should be cautiously considered due to its exemplary nature, it illustrates the potential of AI to contribute to physicians’ decisions in the clinical workflow. Interestingly, a detailed analysis of the false negative images demonstrated that all images where the model failed to predict presence of a fracture corresponded to acute A1 injuries with no apparent dislocation or deformation observed on the X-ray, thus the presence of fracture could only be confirmed via second level imaging. Although elucidating the exact mechanism of the model’s predictions is outside the scope of this work and corresponds to the “black box” dilemma of DL algorithms, one could infer that the model tries to recognize a pattern of a “no fracture” vertebra. When this pattern is lacking, a higher probability of a fracture is then computed.

### 4.2. Choice of DL Model for Fracture Classification Task

Only recently have AI based models been adapted for fracture detection. Chung et al. applied a ResNet-152 convolutional neural model on cropped anteroposterior radiographs of the shoulder to distinguish fractured from normal humeri, achieving an accuracy of 95%, an area under the curve (AUC) of 0.996, sensitivity of 99%, and specificity of 97% [36]. Kim and MacKinnon used a version of the Inception V3 model to identify distal radius fractures on sagittal radiographs, achieving an AUC of 0.954 [37]. Their model analyzed the complete radiograph image instead of a cropped region of interest, as we and most other researchers have done. However, their study was limited by the exclusion of radiographs with single lateral projections inconclusive for presence of fractures, thus eliminating the potential use case for its application in clinical practice. Adams et al. concluded that GoogLeNet achieved a higher overall accuracy (90.6%) compared to AlexNet in predicting presence of femoral neck fractures, also using cropped radiographs. The reference standard was set by confirming the fracture presence intraoperatively, thus cleverly minimizing the bias introduction into the model [35]. Similarly, we minimized the annotation bias by training the model exclusively with radiographs where the presence of fracture was confirmed via CT or MRI data.

A model based on Visual Recognition V3 (IBM, Armonk, NY, USA) was recently used to identify vertebral fractures by Murata et al., achieving an accuracy, sensitivity, and specificity of 86.0%, 84.7%, and 87.3%, respectively [24]. While their results are similar to ours, there are important methodological differences to consider. To avoid the introduction of systematic errors while training the model, all of the fractures included in our study were evaluated individually by expert spinal surgeons before annotation, and then discussed in consensus meetings in case of discrepancy. In contrast, each classifying surgeon in the study by Murata et al. evaluated only a single subgroup of images. While our model was trained to identify anomalies in single vertebrae to eliminate confounding factors and ensure a future clinical applicability—as demonstrated in the heatmap analyses (Figure 7)—Murata’s group analyzed the entire radiograph. The exclusion of cases with multiple traumatic fractures impairs the application of their model in clinical practice. However, the inclusion of anteroposterior radiographs resembles a regular clinical scenario where both projections would be evaluated. In addition to the use of a different model, these factors might have contributed to the marginally better performance achieved in our study.

### 4.3. Clinical Relevance of AI for Automated Traumatic Lesion Detection in Radiographs

The reported rates for missed fractures on TL radiographs in the trauma setting remains high [13,14,16]. Plain radiographs of the spine are commonly the first step performed in trauma routine imaging [7,8]. They provide insights into the nature of the injury, the involvement of one or more spinal columns, and can be used to assess the need for more advanced imaging [40]. Whereas in most high-income countries CT and or MRI are typically performed as next step in the diagnosis routine, these are not readily available in the emergency setting in many low- and middle-income countries. In these countries, the use of plain radiographs to rule out vertebral fractures might be far more widespread than reported [10,11,12]. Additionally, and independently from economic factors, a validated decision aid to determine which trauma patients warrant TL spine imaging does not exist [14]. This controversy is highlighted by the seemingly opposing recommendations of various health institutions. The Eastern Association for the Surgery of Trauma and the American College of Radiology recommend CT as the modality of choice [41]. In contrast, the Advanced Trauma Life Support (ATLS) and the National Institute for Health and Care Excellence recommend plain radiographs as initial imaging modality [7,8]. Some centers have applied a stepwise approach using only plain radiographs to clear the thoracic and lumbar spine, recurring to contrast-enhanced CT only in cases where deformity or pain were present among the other parameters of hemodynamical instability [9]. Despite its current widespread use, false negative rates of up to 67% in diagnosing TL fractures have been reported [13,14,15,16]. Delayed or missed diagnosis are qualified as diagnostic errors by the Institute of Medicine [42] and carry important legal and clinical implications. Legally, misdiagnoses are the most common source of malpractice claims or litigation [43]. Clinically, missed fractures on radiographs have consequences such as malunion with restricted range of motion, posttraumatic osteoarthritis, and joint collapse [44]. In this context, the use of an AI based diagnostic aid tool that supports clinicians to identify vertebral fractures could reduce the frequency of such undesirable outcomes. A commonly mentioned rebuttal for implementation of AI based algorithms is the so called “black box” problem, where the clinician is blinded to the “reasoning” behind the model’s prediction [43]. Visualization techniques such as heatmaps could improve the acceptance of fracture detection systems in clinical practice.

### 4.4. Limitations

The present study had some limitations. First, some centers favor the use of CT or MRI as initial imaging modality for detection of spine injuries, thus obviating the need for diagnostic aid tools such as our model. Second, the image set had a relatively small size. However, the impact of this limitation was mitigated by performing aggressive image augmentation and taking advantage of models pre-trained on the ImageNet dataset. Third, the aim of this study was to develop a model capable of identifying anomalies correlating with the presence of a fracture in a single radiographic projection. A physician relies on several imaging modalities and clinical findings to establish the diagnosis of a fracture. Future studies should also explore the diagnostic capabilities of AI based algorithms on more radiographic projections and eventually different imaging modalities. Regarding the heatmaps, it should be noted that the activation maps do not necessarily visualize the fracture zone, but rather the zones being more important in determining the output of the classifier, which may not correspond to the fracture itself.

## 5. Conclusions

This study demonstrated that existing DL models can be adapted to accurately identify traumatic vertebral fractures on TL sagittal radiographs, as revealed by the performance obtained by our adapted versions of VGG16 and ResNet18. Specifically, the adapted version of ResNet18 achieved higher sensitivity, accuracy, and lower false negative rate. Its performance was similar to that of other models reported in the literature. Interestingly, the model based its prediction on the same image areas that lead human experts to diagnose a fracture. Our findings suggest that current AI based applications could be optimized to create a diagnostic aid tool, which supports clinicians in identifying vertebral fractures. The implementation of such a tool could reduce the frequency of diagnostic errors and thus improve patient outcomes.

## Figures and Tables

**Table 1:** Disease and procedure codes.

Disease Codes	Procedure Codes
Fracture of thoracic spine	Thoracolumbar instrumentation
Fracture of thoracolumbar spine	Instrumentation lumbar spine
Fracture of lumbar spine	Instrumentation thoracic spine
Vertebra fracture	Osteosynthesis of the spine
Vertebra injury	Spinopelvic fixation
	Kyphoplasty
	Spinal fixation

**Table 2:** Performance comparison of the two deep learning convolutional neural models ResNet18 and VGG16.

	Sensitivity	Specificity	Negative Predictive Value	Accuracy
ResNet 18	0.91	0.89	0.89	0.88
VGG16	0.90	0.83	0.89	0.86

## Data Availability

The datasets generated during and/or analyzed during the current study are available from the corresponding author on reasonable request. In order to comply with the requirements of the Ethical Committee, the image set is not available for request due to data privacy policies.
